# A Model-System to Address the Impact of Phenotypic Heterogeneity and Plasticity on the Development of Cancer Therapies

**DOI:** 10.3389/fonc.2019.00842

**Published:** 2019-08-29

**Authors:** Eric D. Jong, Irina C. W. Chan, Aurora M. Nedelcu

**Affiliations:** Biology Department, University of New Brunswick, Fredericton, NB, Canada

**Keywords:** extracellular pH, phenotypic plasticity, therapy, H2122, metastasis, experimental evolution, selection, microenvironment

## Abstract

The main challenges in developing effective anti-cancer therapies stem from the highly complex and heterogeneous nature of cancer, including the presence of multiple genetically-encoded and environmentally-induced cancer cell phenotypes within an individual. This diversity can make the development of successful treatments difficult as different phenotypes can have different responses to the same treatment. The lack of model-systems that can be used to simultaneously test the effect of therapies on multiple distinct phenotypic states further contributes to this problem. To mitigate these challenges, we suggest that *in vitro* model-systems that consist of several genetically-related but phenotypically distinct populations can be used as proxies for the several phenotypes (including adherent and circulating tumor cells) present in a patient with advanced disease. As proof of concept, we have developed such a model and showed that different phenotypes had different responses to the same challenge (i.e., a change in extracellular pH) both in terms of sensitivity and phenotypic plasticity. We suggest that similar model-systems could be developed and used when designing novel therapeutic strategies, to address the potential impact of phenotypic heterogeneity and plasticity of cancer on the development of successful therapies. Specifically, the effect of a therapy should be considered on more than one cancer cell phenotype (to increase its effectiveness), and both cell viability as well as changes in phenotypic state (to address potential plastic responses) should be evaluated. Although we are aware of the limitations of *in vitro* systems, we believe that the use of established cell lines that express multiple phenotypes can provide invaluable insights into the complex interplay between therapies and cancer's heterogeneous and plastic nature.

## Introduction

Cancer is a complex disease that manifests itself in a multitude of ways, partly due to its diverse genetic and phenotypic nature—both among and within individuals. This diversity can also pose significant challenges to cancer therapies (1–5). For instance, high levels of genetic heterogeneity are known to be associated with more aggressive cancers and resistance to drugs, and distinct phenotypes have been shown to have different responses to therapies and even cooperate to increase their survival or to drive invasion (2, 5–7). Given its genetic, phenotypic, and behavioral complexity, evolutionary and ecological theories are now being applied to understand the processes that affect cancer initiation, progression and dissemination (1, 8–10). Specifically, cancer is seen as part of a complex ecosystem (the body) in which the survival and reproduction of the distinct cancer cell phenotypes can be affected by their local environments (e.g., location in the tumor), changes in the conditions of their specific habitats (tissues), or new selective pressures (such as therapies) (11, 12).

## The Problem

The dynamic interactions between the diverse and continuously evolving genotypes and their fluctuating environments can add difficulty to developing successful therapies. Increased levels of genetic diversity can allow selection for clones with phenotypes that confer adaptive advantage in specific settings, including the presence of various drugs. This unescapable evolutionary aspect of cancer hinders the development of successful therapies as drug resistance is always expected to evolve (13–15). Consequently, an increased body of research has been devoted to understanding the degree of genetic heterogeneity in tumors and predicting patterns of mutation and selection in response to therapies (7, 16). However, specific microenvironmental signals can also alter the phenotype directly by affecting gene expression patterns and inducing alternative phenotypes that are better fit to the new challenges. Phenotypic plasticity is most relevant during metastasis and dissemination stages, when cells undergo transitions from epithelial to mesenchymal phenotypes (and back), and express distinct cell behaviors [e.g., adherent or invasive cells, circulating tumor cells (CTC), or disseminated cells] specific to different progression stages (localized or advanced disease) and cell fates (epithelial, mesenchymal, cancer stem cells) (17, 18). Such cell plasticity has a strong impact on therapies as phenotypic changes and the presence of multiple phenotypes can drastically reduce the effectiveness of treatments (19–21). For instance, shifts in the proportion of epithelial and mesenchymal cells among CTCs have been found to accompany cycles of response to therapy and disease progression in breast cancer (21). Similarly, epithelial-to-mesenchymal transition (EMT) has been shown to mediate resistance to the drug Docetaxel in prostate cancer (22). CTCs, and especially CTC clusters, are also generally more resistant to chemotherapy, possibly due to lack of proliferation and their enrichment in mesenchymal cells (23–25) or cell detachment per se (by mechanisms independent of EMT) (26). Consequently, drugs and therapies directed at targeting specific phenotypes, or blocking cellular plasticity are now being considered (3, 4, 27).

Despite the diversity of genetically-encoded and environmentally-induced cancer cell phenotypes within an individual, most *in vitro* studies directed at developing new therapeutic strategies generally employ established cell lines with an adherent phenotype [either as monolayers or 3D cancer spheroids (26, 28)]. Nevertheless, efforts are being made toward using additional phenotypes. For instance, patient-derived CTCs have been used to establish *in vitro* cultures for personalized drug sensitivity tests (29, 30); however, such systems might not fully capture the behavior of real CTCs or CTC clusters (31) and cannot address the range of responses that other phenotypes in a patient might express. More importantly, most studies addressing the effect of drugs and micro-environmental changes generally consider only responses in terms of cell survival and proliferation, without considering possible changes in the phenotypic state of cancer cells (32). This situation is partly due to the lack of model-systems that can be used to simultaneously investigate the response of different cancer cell phenotypes specific to different progression stages, especially the dispersal stage involving CTCs.

## An *in vitro* Model-System to Capture Cancer's Heterogeneity

**Figure 1:**
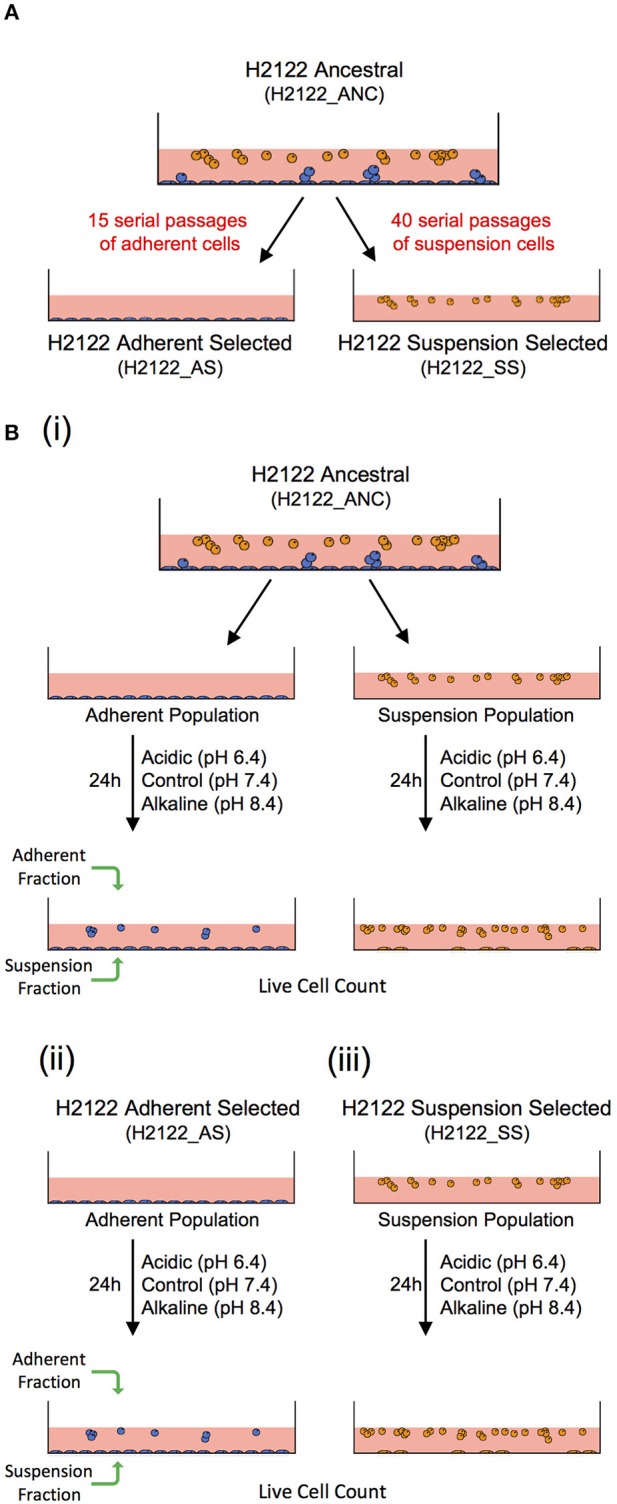
An *in vitro* model-system to address the effect of the same microenvironmental change on genetically-related but phenotypically distinct cancer cell populations. **(A)** A non-small cell lung cancer line (H2122_ANC) was used to select for an adherent line (H2122_AS) and a suspension line (H2122_SS) through 15 and 40 serial passages of only the adherent or suspension populations, respectively. **(B)** Experimental design to address the effect of extracellular acidic and alkaline pH on the ANC_Adherent and ANC_Suspension populations of the (i) ANC line, and the (ii) Adherent Selected (AS) and (iii) Suspension Selected (SS) lines.

To this end, we suggest that *in vitro* model-systems that could be used to address the effect of the same microenvironmental change or selective pressure on different cancer cell phenotypes (adherent—such as in tumors, and in suspension—similar to CTCs and CTC clusters) sharing a related genetic background can be developed. Here, we describe the development of such a model. Specifically, we started with a non-small cell lung cancer line (NCI-H2122 [H2122] (ATCC® CRL5985™); ATCC) that was initially derived from a metastatic site (pleural effusion) of a stage 4 adenocarcinoma lung cancer patient. The line grows as a mixture of adherent cells (with round and epithelial morphology) and cells in suspension—both as single cells and grape-like clusters that are loosely adherent or remain in suspension. This cell line (from here on referred to as the ancestral, or ANC line) was used to experimentally evolve two additional lines: one line selected for adherent growth (referred to as the Adherent-Selected or AS line) and one line selected for growth in suspension (referred to as the Suspension-Selected or SS line), by selectively passaging only the adherent or the suspension cell populations, respectively (Figure 1A).

These three lines are genetically related but phenotypically distinct, and thus could be used to address the effect of the same microenvironmental challenge on multiple cancer cell phenotypes. Because the ANC line consists of two distinct populations (adherent and suspension) that express phenotypic plasticity (i.e., adherent cells can detach, and cells in suspension can attach), this line could be useful to test the effect of the same challenge on systems that comprise multiple phenotypes, such as during advanced disease. On the other hand, the AS line is strictly adherent (like most established cell lines used in cancer research) and, functionally, can be viewed as analogous to a non-disseminating tumor (i.e., cells do not have the potential to detach). Lastly, as the clusters in both the ANC and SS lines share a series of features with real CTC clusters, including cluster size, morphology, cell-cell connections and the expression of both epithelial and mesenchymal markers (31), these lines can be valuable model-systems to investigate the biology of CTC clusters and test potential therapeutic strategies specifically directed against them.

## Proof of Concept

As proof of concept that this model can be used to test the effect of the same challenge on a system comprising genetically-related but phenotypically distinct populations with potential for phenotypic plasticity, we investigated the response of the lines described above to changes in extracellular pH (pHe), in terms of both overall sensitivity and phenotypic plasticity (i.e., the ability of cells to detach and survive in suspension or to attach). Extracellular pH was chosen as the experimental variable because it is known to affect cancer cell behaviors and metastatic potential of tumors *in vivo* (33); for instance, acidic pHe (around 6.4) promotes metastasis (34), whereas strategies that employ buffers to restore pH to the normal range can suppress this process (35, 36). Recent *in vitro* studies also showed a reduction in cell adhesion (paralleled by changes in the expression of EMT markers) in two cancer cell lines exposed to acidic pH (6.6) for 48 h (37). Nevertheless, the two pH levels used in this study were chosen arbitrarily as 1 unit below and above the pH of the growth medium, simply as a means to induce measurable non-lethal environmental alterations that can cause clear and quantifiable phenotypic changes (i.e., cell detachment/attachment).

Cultures were maintained in RPMI-1640 medium (ATCC) supplemented with 10% fetal bovine serum (ATCC) and 1% GIBCO™ Penicillin-Streptomycin (10,000 U/ml), from here on referred to as RPMI. Acidic and alkaline media were prepared by adjusting the pH of RPMI (pH 7.4) with 1M HCl (to pH 6.4) and 1M NaOH (to pH 8.4), respectively. For the ANC line, 2.0 × 10^5^ cells were seeded in individual wells of a 12-well plate and grown in regular RPMI overnight, to allow the adherent population to attach to the substrate before the experimental treatments. Next day, the suspension cell population (ANC_Suspension population) was removed from these wells and re-suspended in media at pH 6.4 (acidic), 7.4 (control), or 8.4 (alkaline), and then placed into new wells. The cells adherent to the wells (ANC_Adherent population) were also subjected to the same three media. For the AS cell line, 2.0 × 10^5^ cells per well were seeded in RPMI, allowed to adhere overnight and then the medium was replaced with either control, acidic or alkaline media. For the SS line, 2.0 × 10^5^ cells were seeded in individual wells containing media at each of the three pH levels. Twenty-four hours later, the suspension and adherent cell fractions from each well were collected separately and the number of live cells in each fraction was assessed (Figure 1B). The total numbers of live cells (irrespective of phenotype) in each of the four populations, as well as the numbers of live cells for each fraction/phenotype separately, were compared among the three pH group levels using a one-way ANOVA; Dunnett's multiple comparisons test was used to evaluate significant differences (*p* < 0.03) between each treatment and the control (GraphPad Prism7).

### Different Phenotypes Respond Differently to the Same Set of Challenges

**Figure 2:**
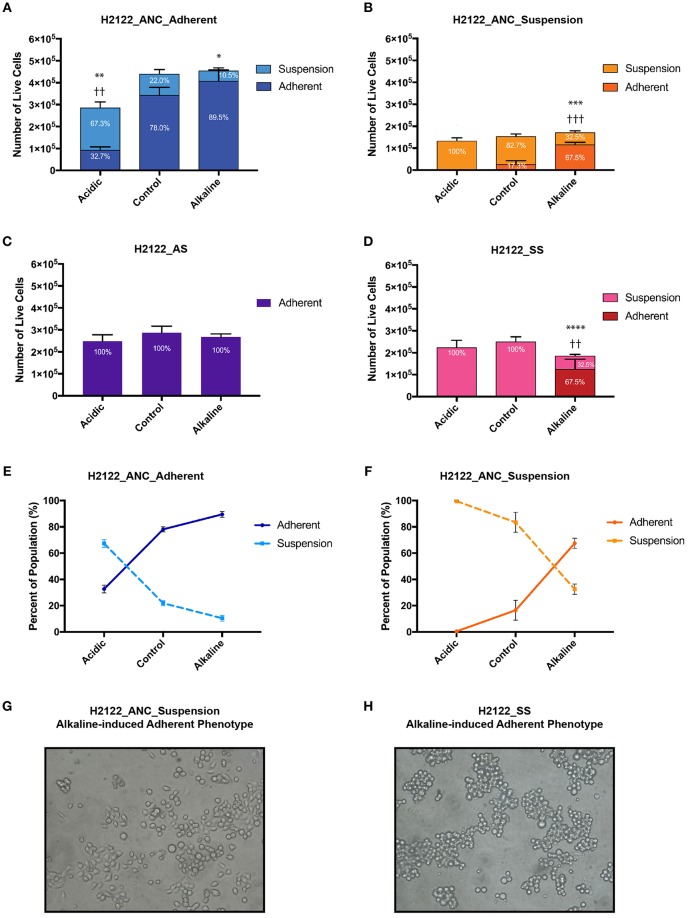
The effect of extracellular pH on the size (as number of live cells) and phenotypic composition (as percentage of live cells in the adherent and suspension cell fractions relative to the entire population) of four genetically-related but phenotypically distinct cancer cell populations subjected for 24 h to acidic (pH 6.4), control (pH 7.4), and alkaline (pH 8.4) media. **(A)** H2122_ANC_Adherent Population. **(B)** H2122_ANC_Suspension Population. **(C)** H2122_AS line. **(D)** H2122_SS line. **(E,F)** Changes in the proportion of the suspension and adherent fractions in the Adherent and Suspension populations of the ANC line, as a function of pH [data are from **(A,B)**]. **(H)** Attached H2122_SS cells exhibit round morphology following treatment with alkaline media; **(G)** Attached H2122_ANC_Suspension cells exhibit a mixture of round and epithelial-like morphology following treatment with alkaline media. All treatments were performed in 1.5 ml media, with 3 biological replicates. Viability was assessed using SYTO 9 and Propidium Iodide (PI) (Molecular Probes) and the Countess II FL Automated Cell Counter (ThermoFisher Scientific). SYTO 9 stains DNA in both dead and live cells, while PI is only permeant to necrotic and late apoptotic cells. Because this method could miss early apoptotic cells with intact membranes and apoptotic cells that disintegrated, numbers of dead cells are not reported here. All data were expressed as means ± SD of 3 biological replicates (each with 3 technical replicates). ^*^ and ^†^ indicate significant differences in the numbers of live cells of the suspension and adherent fractions, respectively, between treatments and control (^*^p < 0.0332, ^**^/^††^*p* < 0.0021, ^***^/^†††^*p* < 0.0002, ^****^p < 0.0001). Total population sizes did not show statistically significant differences, except in one case (see text).

We subjected each of the four cancer cell populations that share a related genetic background but express distinct phenotypes (i.e., ANC_Adherent, ANC_Suspension, AS, and SS) to media of three pH levels—acidic, control, and alkaline, and assessed differences in overall population size and phenotypic state (adherent vs. suspension; Figure 1B). We found that although changes in pH levels did not have a strong effect on the overall size of the populations (with the exception of the acidic treatment of the ANC_Adherent population; Figure 2A; *p* < 0.001), the acidic and alkaline treatments drastically affected the phenotypic composition (relative to the control pH) of three of the four populations (Figure 2). In the ANC_Adherent population, acidic pH resulted in a higher percentage of suspension cells (3-fold difference, relative to the control), whereas in the alkaline pH treatment, the percentage of suspension cells was half of that in the control (Figure 2A). In the ANC_Suspension population, the adherent fraction was completely lost in the acidic environment, but in the alkaline media the percentage of adherent cells was almost 4-time higher relative to the control (Figure 2B). Lastly, while the AS line was unaffected by either treatment (Figure 2C), the SS line recovered its ability to adhere in the alkaline environment (Figure 2D). Interestingly, in contrast to the epithelial-like morphology expressed by most alkaline-induced adherent cells of the ANC_Suspension population, most SS adherent cells exhibited a round morphology (Figures 2G,H).

Generally, the four populations displayed substantial heterogeneity in response to the two pH challenges: the phenotypic composition of the adherent and suspension populations of the ANC line was affected by both acidic and alkaline pHe, the SS was only affected by the alkaline pHe, and the AS appeared unaffected by both treatments. Also, the same challenge had different effects on the four populations. Acidic pH affected the phenotypic composition of both the adherent and suspension populations of the ANC line (and also affected the size of the ANC_Adherent population), but had no effect on the phenotypic composition of the AS and SS populations. On the other hand, alkaline media affected both the adherent and suspension populations of the ANC line as well as the SS line, while the AS line was unaffected. Taken together, these findings argue for the potential benefit of testing the effect of therapies on different phenotypes using systems similar to the one we developed.

### Phenotypic Plasticity Underlies pH-induced Differences in Phenotypic Composition

Because of the short length of the treatment (24 h) and the doubling time of this cell line (estimated as at least 48 h), the drastic differences in the proportion of phenotypes we observed (in most cases without differences in the overall population size) can only be explained by invoking phenotypic switches. For instance, relative to their controls, in both ANC populations (ANC_Adherent and ANC_Suspension), acidic pH resulted in a higher proportion of cells in suspension (paralleled by a lower proportion of adherent cells; Figure 2E). At least for the ANC_Adherent population (that shows a much larger number of cells in suspension relative to the control), this difference can only be accounted for by increased cell detachment in the acidic environment relative to its control. Similarly, alkaline pH had the opposite effect on both populations—it caused a higher proportion of the adherent phenotype at the expense of the suspension phenotype (Figure 2F). Again, at least for the ANC_Suspension population in the alkaline environment, the higher number of adherent cells (relative to its control) can only be explained by a large number of suspension cells assuming an adherent phenotype. Such pH-induced changes in phenotypic states are further supported by the behavior of the SS line, which is phenotypically homogeneous in the control medium, but becomes heterogenous in the alkaline medium, with a large proportion of cells being adherent (Figure 2D).

Overall, we suggest that this model-system can also be used to gain a better understanding of potential phenotypic switches in response to microenvironmental changes. As the behavior of both ANC populations is consistent with previously known responses to pH *in vivo* [i.e., acidic tumor environments promote metastasis while neutralization can suppress it; (35)], our findings argue that this line could be useful as a proxy for metastatic stages, in which the phenotypic state of cells (especially with respect to cell detachment and cell survival in circulation) is likely to be affected by changes in their microenvironments. On the other hand, the AS line did not exhibit plasticity, providing a complementary phenotypic state to the ANC line, which can allow investigations into potentially dissimilar responses of genetically-related but phenotypically distinct populations.

## Future Directions

The specific goal of this Perspective is 3-fold: (i) to argue that the use of *in vitro* model-systems that comprise genetically-related but phenotypically distinct cancer cell populations can help capture the phenotypic landscape in a patient with advanced disease, (ii) to highlight the different responses exhibited by different cancer cell populations that are subjected to the same microenvironmental challenge, in terms of both their overall sensitivity and phenotypic state, and (iii) to suggest that these model-systems can be used to both test the effect of therapies on more than one phenotype (to ensure effectiveness and avoid unexpected effects) and design therapies specific for different phenotypes (to improve their effectiveness).

As proof of concept that such lines can be valuable and useful complementary systems for testing and developing new therapies, we used media of two different pH levels and investigated the response of four genetically-related but phenotypically distinct populations. Our data showed clearly that not all phenotypes are sensitive to the same challenge, and that the same challenge can have different effects on both the sensitivity and the phenotypic composition of distinct populations. We believe these findings provide a strong case for the usefulness of such model-systems to investigate the ways in which therapies can affect the range of cancer cell phenotypes likely to be present in the same individual. This would both increase the effectiveness of therapies (as different phenotypes might show different sensitivities to the same treatment) and address potential negative effects (by inducing alternative phenotypes). For instance, the same challenge (e.g., low pHe in our study) that may induce an apparent decrease in the size of a population (as in the ANC_Adherent population; Figure 2A) might have no effect on other phenotypically different populations (the ANC_Suspension population, AS and SS lines; Figures 2B–D). More importantly, although a therapy might result in the decline or stabilization of a cancer population, its phenotypic composition can change in significant ways. For instance, while a therapy may decrease the size of a population (as low pHe did for the ANC_Adherent population), it may also induce the detachment of live cells (Figure 2A; note that the acid-induced suspension fraction proliferated and expressed a mixture of both phenotypes when re-cultured in the three pH media; data not shown). Similarly, a treatment (in our case, alkaline media) that might repress cell detachment (the ANC_Adherent population; Figure 2A) may also promote the adherence of cells already in circulation (the SS line; Figure 2D) and possibly facilitate the colonization stage. Lastly, during systemic therapies, a therapeutic agent can reach different levels in distinct regions of the body (bloodstream vs. tumor); this can have different effects in terms of the cell phenotype induced: at high levels it can promote one phenotype (and inhibit the alternative state) while at low levels can have the opposite effect (Figures 2E,F).

Although here we used pH as a proxy for microenvironmental changes, other factors are likely to have similar differential effects on the sensitivity and phenotypic plasticity of cancer cell populations. For instance, we found that the four cell populations in our model-system have different sensitivities to glucose deprivation, with the SS line being most resistant to glucose deprivation (our unpublished data). Similar findings have been reported for two sublines (with distinct phenotypes) derived from a small cell lung line (NCI-H69); the adherent subline showed increased resistance to etoposide, cyclophosphamide, and gamma radiation when compared to the original line growing in suspension (38, 39). Regarding phenotypic plasticity, we found that glucose deprivation alone or in combination with metformin [an anti-diabetic drug with potential anti-cancer activities; (40)] induces cell detachment in the H2122_ANC and AS lines (our unpublished data). Similar responses have been also reported in two breast cancer lines as co-treatment with 2-deoxy-D-glucose (used to mimic glucose starvation) and metformin resulted in the detachment of viable cells (32).

The model-system we presented here used an established non-small lung cancer cell line that we experimentally evolved into two additional lines. The ANC line is highly heterogeneous, containing a mixture of cells expressing one of two stable phenotypes (strictly adherent or strictly suspension) as well as cells expressing some level of phenotypic plasticity (being able to express both phenotypes). Its heterogeneity and plasticity could be valuable in addressing the effect of therapies on multiple plastic phenotypes. The cell clusters in this line are also heterogeneous. They consist of a mixture of cells expressing epithelial or mesenchymal markers—a feature shared with CTC clusters isolated from cancer patients, and thus could be used in developing therapies directly targeted against CTC clusters (31).

Many other cancer cell lines available from ATCC (of lung, breast, prostate, pancreatic, colon, gastric origin) grow as mixtures of adherent cells and cells in suspension, and thus can be used as proxies for genetically heterogeneous and phenotypically plastic tumors with dispersal potential. Selection experiments similar to the ones we presented here can also be performed on such lines to evolve specific phenotypes. In fact, a small cell lung cell line (NCI-H69) that grows mostly as tightly-packed floating aggregates has been previously used to derive an adherent subline. The suspension and adherent lines were shown to differ in their sensitivity to drugs (discussed above) as well as expression of EMT markers, with the ancestral suspension line being positive for E-cadherin while the derived adherent line was negative for E-cadherin and positive for vimentin (39). Many other established cell lines that grow strictly (or mainly) as cell clusters in suspension are also available from ATCC, and they can be used to identify potential targets that are common to the CTC cluster phenotype (31).

## Conclusions

Based on the growing evidence regarding the impact of phenotypic heterogeneity and plasticity on the success of current therapies (discussed in the Introduction) we argue that alternative model-systems that can better capture cancer's heterogeneity and plasticity could be increasingly integrated in the development of new therapeutic strategies, in three important ways. First, since responses to therapies are known to differ among cancer cell phenotypes (with some phenotypes—such as CTCs and CTC clusters being more refractory to standard treatments), model-systems expressing heterogeneous phenotypes could assist in the development of phenotype-specific therapies. Second, changes in phenotypic state should also be evaluated to address potential unforeseen effects. Third, to increase the efficiency of therapies, strategies directed at altering the tumor microenvironment [such as altering pHe; (36, 41)] to prevent phenotypic transitions could be considered; model-systems like the one we developed can help identify such strategies. Although we are aware of the limitations of *in vitro* models (including the system we presented here), we believe that the use of experimental evolution and established cell lines that express phenotypic plasticity can provide invaluable general insights into the complex responses that therapies can trigger in a cancer patient.

## Data Availability

All datasets generated for this study are included in the manuscript.

## Author Contributions

EJ designed and performed research, acquired, analyzed and interpreted data, and contributed to the writing of the manuscript. IC designed and performed research, acquired, analyzed and interpreted data, and edited the manuscript. AN designed research, interpreted data, wrote the manuscript, and supervised the study.

### Conflict of Interest Statement

The authors declare that the research was conducted in the absence of any commercial or financial relationships that could be construed as a potential conflict of interest.
